# 

**Published:** 1911-08

**Authors:** 


Vol. XXVII. AUGUST, 1911. No. 2

### TEXAS MEDICAL JOURNAL

INDEXED

Established
July, 1885

"Red Back"

PUBLISHED AT AUSTIN, TEXAS, BY

F. E. DANIEL, M. D., - - - Editor and Proprietor.

PRINTED BY THE VON BOECKMANN-JONES CO.

Subscription, $1.00 a Year, in Advance. Single Copies, 10 Cents

